# Supplementary material for: Electron counting detectors in scanning transmission electron microscopy via hardware signal processing
Source: Nat Commun. 2023 Aug 25;14:5184. doi: 10.1038/s41467-023-40875-w (PMC10457289; doi:10.1038/s41467-023-40875-w)
Supplement: Supplementary file 1 — Supplementary Information [file 41467_2023_40875_MOESM1_ESM.pdf]

Supplementary materials for Electron counting detectors in scanning transmission electron microscopy via hardware signal processing

Jonathan J. P. Peters[1,2], Tiarnan Mullarkey[1,3], Emma Hedley[4], Karin H. Muller[5], Alexandra Porter[5], Ali Mostaed[4], Lewys Jones[1,2,3]

[1] Advanced Microscopy Laboratory (AML), Trinity College Dublin, the University of Dublin, Dublin, Ireland
[2] School of Physics, Trinity College Dublin, the University of Dublin, Dublin, Ireland
[3] Centre for Doctoral Training in the Advanced Characterisation of Materials, AMBER Centre, Dublin, Ireland
[4] Department of Materials, University of Oxford, Oxford, United Kingdom
[5] Faculty of Engineering, Department of Materials, Imperial College London, London, United Kingdom

Detector Imperfections

The detector imperfections (flatness, roundness, ellipticity, and smoothness) vary between individual detectors, even within the same manufacturer/model, and can be affected by the use of the detector (e.g. over-exposure and damage). In Fig. S1 we present measurements from a range of detectors and a range of different manufacturers. All detectors except for detector I are based on scintillators, with detector I being a silicon based solid-state detector.

In calculating the flatness of the detector, the inner and outer angle of the detector must be defined. In this work, this is defined as follow. First a binary mask of the 2D detector image is defined as any area in the counted image with an intensity greater than the midpoint of the dynamic range. This binary mask is then azimuthally averaged, and the active region is defined as the components with values greater than 0.9. This fraction is somewhat arbitrary, though using a lower threshold for the azimuthally averaged mask will include effects of ellipticity or non-concentricity.

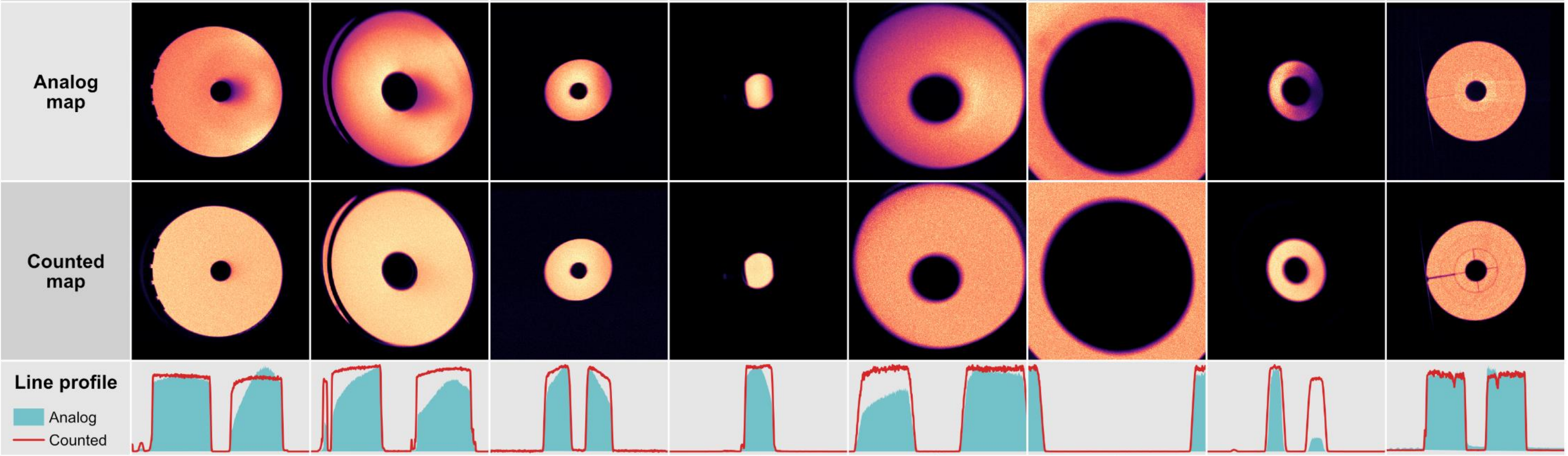

	Company 1		Company 2						Company 3				Company 4		Company 5	
	Detector A		Detector B		Detector C		Detector D		Detector E		Detector F		Detector G		Detector I	
	Analog	Counted	Analog	Counted	Analog	Counted	Analog	Counted	Analog	Counted	Analog	Counted	Analog	Counted	Analog	Counted
Flatness	9.86 %	2.20 %	6.69 %	1.88 %	9.61 %	4.07 %	8.97 %	1.91 %	8.38 %	2.27 %	4.07%	1.74 %	21.60 %	5.94 %	2.94 %	2.28 %
Roundness	5.08 %	1.62 %	5.42 %	0.87 %	6.63 %	2.56 %	6.27 %	0.67 %	15.95 %	1.33 %	5.77 %	1.93 %	49.64 %	4.67 %	1.34 %	2.70 %
Smoothness	29.77 %	14.83 %	51.76 %	16.90 %	42.55 %	21.88 %	51.19 %	23.65 %	58.50 %	26.09 %	33.16 %	24.82 %	149.81 %	31.08 %	22.62 %	23.64 %
Ellipticity	1.88 %		12.37 %		2.81 %		†25.84 %		4.90 %		1.88 %		10.69 %		2.82 %	

Figure S1 Full detector imperfection measurements Detector imperfections measured for 8 commercially available electron detectors from 5 manufacturers. Analog and counted maps were acquired simultaneously. Note that the ellipticity for detector D (marked with a †) uses the outer radius where all other measurements use the inner radius.

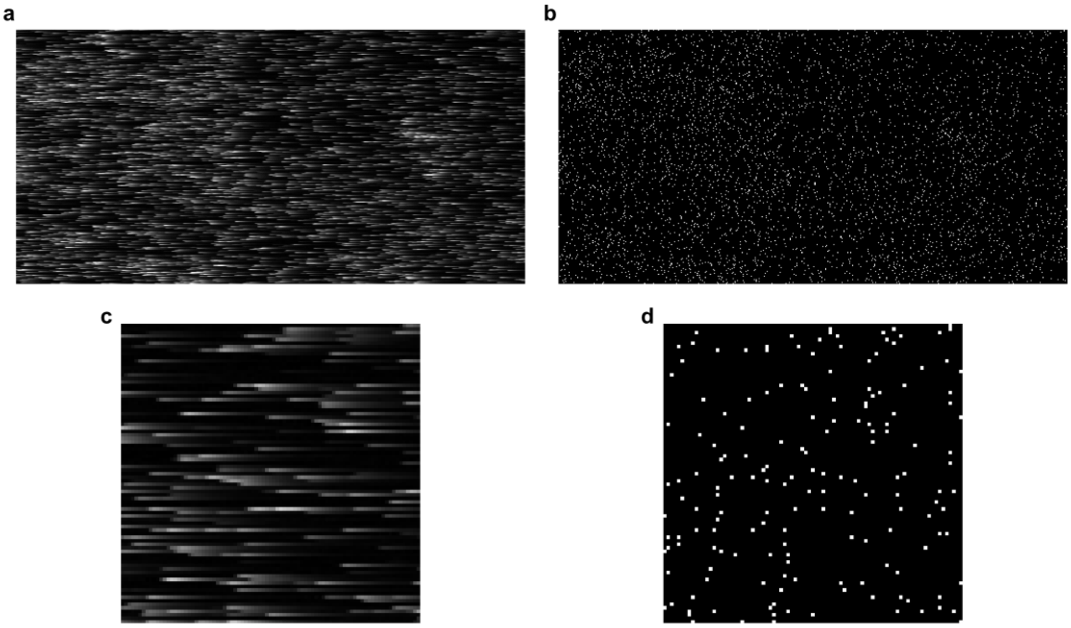

Figure S2 Low dose pulse streaking a Single frame analog image a the cell wall of a human macrophage showing the same region as Fig. 4b in the main text. Image is 256×512 pixels with 50 ns per pixel. **b** Counted image acquired simultaneously to **a**. **c**, **d** Zoomed in regions of a and b, respectively, taken from the bottom right corner of each image.

Low dose temporal response

The slow temporal response in analog imaging is clearly seen in low dose images, where the number of electron detection events is below 1 per pixel. Such an example images is shown in Fig. S2 with both analog and counted acquisition. The electron pulse shape of Fig. 1d in the main text is clearly visible across multiple pixels.

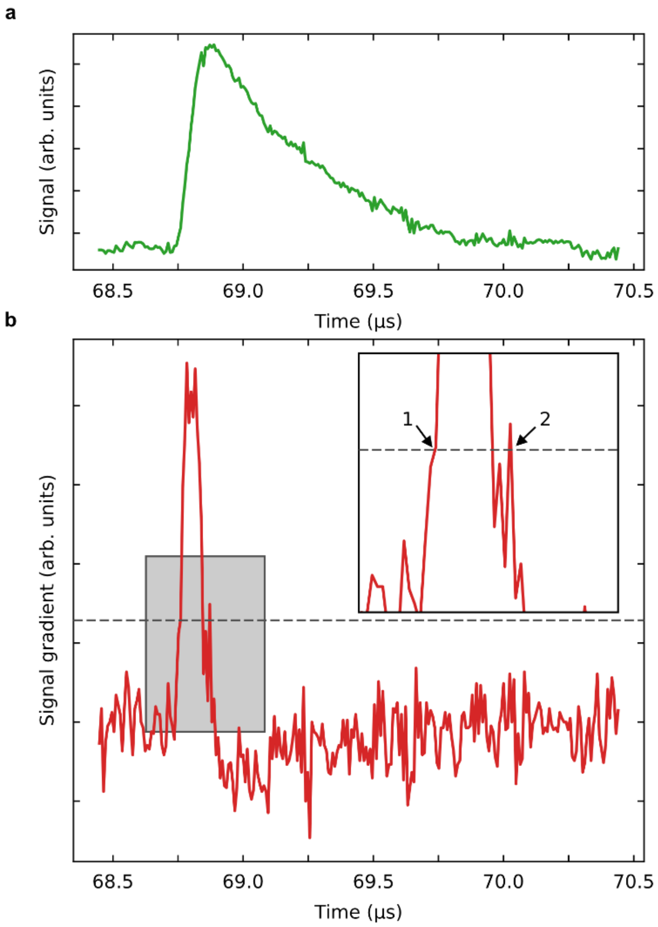

Figure S3 Threshold noise rejection a Example electron pulse. **b** Signal gradient showing a case where multiple electrons can erroneously be detected for a given threshold (shown in dashed line). Inset is a zoomed in region of the grey box. The points at which electrons are detected are labelled 1 and 2.

Noise rejection

Depending on the detector, control electronics, and environment, the detector signal quality may be degraded. For lower quality signals, it is possible to erroneously detect noise as electrons. An example is shown in Fig. S3 where a relatively well-defined pulse (Fig. S3a) gives a gradient signal that can be thresholded such that 2 electrons can be detected (inset in Fig. S3b). A simple yet effective approach to reject signal noise is to require that the thresholding of the signal gradient must remain above the threshold for a set period before an electron is registered. Typically, this is only required to be 16 or 24 ns (2 or 3 samples for the hardware shown here). This also has the benefit that the digital output when an electron is detected is not significantly delayed.

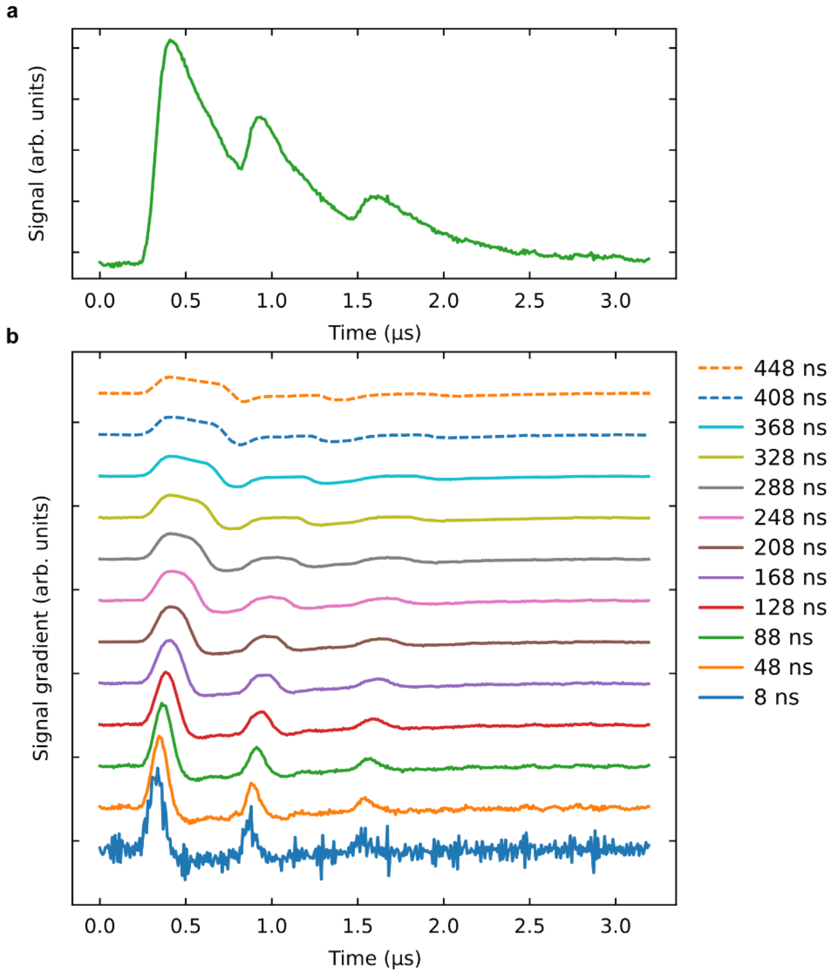

Figure S4 Signal gradient step a Example detector signal showing 3 electron pulses. **b** Gradient calculation of the signal in **a** with different time steps when calculating the signal.

It is possible to choose a time step to calculate the gradient to further reject noise. For example, when sampling the signal at a high frequency, the difference between neighbouring samples on a rising edge is often not significantly above the noise level, however, across a number of samples the rising edge is clear. The gradient step should be tuned to the specifics of each detector system such that the signal gradient is clear, but rapid events can be distinguished. Figure S4 shows an example data stream with 3 clear pulses. By increasing the gradient step (Fig. S4b) noise can be reduced, but also the gradient signal can be attenuated. Increasing the gradient step also increase the lag between the actual electron detection event and the digital output, so should be as small as possible.

Sample preparation

Human monocyte

For TEM, Human monocyte derived macrophage cells (HMMs) were grown on 6-well tissue culture plates, incubated with graphene (at a concentration of 10µg/ml) for 4 hours in SFM culture medium at 37 °C. After the incubation period, cell monolayers were washed twice in 0.9 % saline to remove any non-ingested particles and subsequently fixed with 4 % glutaraldehyde (in 0.1 M HEPES buffer, pH 7.2) for 1 h at 4 °C. Cells were scraped using a cell scraper and washed several times in deionized water (DIW) to remove fixative. Then, samples were osmicated (1% OsO_4, 0.15 % potassium ferricyanide; 2 mM $CaCl_2$ in DIW) for 1 h at RT. Again, samples were washed several times with DIW and then bulk stained for 1 h at RT in the dark using uranyl acetate. Following 2 washes in DIW, the samples were dehydrated in graded solutions of ethanol (70 %, 95 %, 100 %), 3x in each for 5 minutes, respectively. After two additional washes in 100 % acetonitrile, samples were infiltrated with Quetol 651 resin over 5 days using fresh resin each day. Resin was cured at 65 °C for 48h. Ultrathin sections (~ 70nm) were cut using a Leica Ultracut ultramicrotome, mounted on 300 mesh bare copper grids.

$LaFeO_3$

The lanthanum ferrite ceramic was prepared from La_2O_3 and Fe_2O_3 powder through a solid-state reaction process. Further information is presented by A. Mostaed et al. (2021).[1] To achieve electron transparency, samples were thinned using Ar ions in a Gatan PIPS.

Gold nanoparticles

We used high resolution combined test specimen containing gold particles on carbon film purchased from Agar Scientific.

$SrTiO_3$

(100) oriented $SrTiO_3$ substrates were commercially purchased, from which a TEM lamella sample was prepared. A Tescan Amber focussed ion beam (FIB) was used with standard FIB lift-out techniques.

Supplementary References

1. Mostaed, A. *et al.* Characterizing oxygen atoms in perovskite and pyrochlore oxides using ADF-STEM at a resolution of a few tens of picometers. *Acta Mater* 208, 116717 (2021).
